# Nursing support for testicular sperm aspiration and intracytoplasmic sperm injection in patients with high paraplegia: a retrospective case series

**DOI:** 10.1186/s12610-025-00281-2

**Published:** 2025-09-02

**Authors:** Qun Wei, Xia Hong, Yu He, Zilian Wang

**Affiliations:** Nursing Department, College of Medicine, Sir Run Run Shaw Hospital, Zhejiang University, Qingchun East Road No.3, Hangzhou, Zhejiang Province 310016 People’s Republic of China

**Keywords:** High paraplegia, Testicular sperm aspiration, Intracytoplasmic sperm injection, Assisted reproductive technology, Nursing care interventions, Paraplégie élevée, Aspiration testiculaire de Spermatozoïdes, Injection intracytoplasmique de Spermatozoïdes, Technologie de Procréation assistée, Interventions en Soins infirmiers

## Abstract

**Background:**

High paraplegia, primarily affecting young men during their reproductive years, often results in ejaculatory dysfunction and infertility. Testicular sperm aspiration (TESA) combined with intracytoplasmic sperm injection (ICSI) offers a viable path to biological parenthood for this population. However, evidence on nursing strategies supporting such procedures is limited. This study aimed to evaluate the clinical and psychosocial outcomes of patients with high paraplegia due to spinal cord injury who underwent TESA-ICSI and explore the effectiveness of specialized nursing interventions.

**Case presentation:**

A retrospective case series was conducted on nine male patients with high paraplegia treated at Sir Run Run Shaw Hospital (Zhejiang, China) between January 2021 and October 2023. The mean age of the patients was 31.89 ± 4.83 years, with injury durations ranging from 1 to 13 years. Psychological evaluations using the SAS, SDS, SSRS, and WHOQOL-BREF scales revealed that three patients had anxiety, four had depression, and most had moderate to high social support. The WHOQOL-BREF scores indicated generally good perceived quality of life (score range, 82–91). TESA was performed under local anesthesia, with perioperative nursing measures including bladder management, wound care, and infection prevention. Viable sperm were successfully retrieved in all cases without complications. Eight couples proceeded to ICSI, with viable embryos obtained in all. Six couples achieved pregnancy (66.7%)and live births. One case ended in miscarriage due to trauma, and one couple had no embryos suitable for transfer.

**Conclusion:**

Comprehensive nursing interventions, including psychological support, ethical evaluation and procedural adaptations, may facilitate the successful application of TESA-ICSI in men with high paraplegia, highlighting the need for standardized interdisciplinary nursing protocols to optimize fertility outcomes in this underserved population.

## Résumé

**Contexte:**

Une paraplégie haute, affectant principalement les jeunes hommes pendant leurs années de reproduction, entraîne souvent une dysfonction de l’éjaculation et une infertilité. L’aspiration testiculaire de spermatozoïdes (TESA) combinée à l’injection intracytoplasmique de spermatozoïdes (ICSI) offre une voie viable vers la parentalité biologique pour cette population. Cependant, les données probantes sur les stratégies infirmières à l’appui de ces procédures, sont limitées. Cette étude visait à évaluer les résultats cliniques et psychosociaux des patients atteints de paraplégie haute due à une lésion de la moelle épinière, qui ont subi une TESA-ICSI, et à explorer l’efficacité des interventions infirmières spécialisées.

**Présentation des Cas:**

Une série rétrospective de cas a été menée sur neuf patients masculins atteints de paraplégie haute traités à l’hôpital Sir Run Run Shaw (Zhejiang, Chine) entre janvier 2021 et octobre 2023. L’âge moyen des patients était de 31,9 ± 4,8 ans, avec des durées de lésion allant de 1 à 13 ans. Des évaluations psychologiques à l’aide des échelles SAS, SDS, SSRS et WHOQOL-BREF ont révélé que trois patients souffraient d’anxiété, quatre de dépression, et la plupart bénéficiaient d’un soutien social de modéré à élevé. Les scores WHOQOL-BREF indiquaient généralement une bonne qualité de vie perçue (plage de scores, 82–91). La TESA a été réalisée sous anesthésie locale, avec des mesures de soins infirmiers per-opératoires comprenant la gestion de la vessie, le soin des plaies et la prévention des infections. Des spermatozoïdes viables ont été récupérés avec succès dans tous les cas, sans complications. Huit couples ont procédé à l’ICSI, avec des embryons viables obtenus pour tous. Six couples ont obtenu une grossesse (66,7%) avec accouchement d’enfants vivants. Un cas s’est terminé par une fausse couche due à un traumatisme, et un couple n’a pas eu d’embryons propices au transfert.

**Conclusion:**

Des interventions infirmières complètes, y compris le soutien psychologique, l’évaluation éthique et les adaptations procédurales, peuvent faciliter l’application réussie de TESA-ICSI chez les hommes atteints de paraplégie haute, soulignant la nécessité de protocoles infirmiers interdisciplinaires standardisés pour optimiser les résultats de la fertilité dans cette population défavorisée.

## Background

High paraplegia, which can be considered as paraplegia resulting from spinal cord injury (SCI) above the second thoracic vertebra, is characterized by the complete loss of motor and sensory function below the level of injury, often accompanied by impaired bladder and bowel control [1]. The global incidence of SCI varies based on trauma-related and non-traumatic causes. A recent systematic review and meta-analysis estimated the overall incidence rate at 23.77 per million people, with traumatic SCI (TSCI) at 26.48 per million and non-traumatic SCI (NTSCI) at 17.93 per million [2]. Notably, approximately 80% of SCI cases occur in young men during their reproductive years [3], and in this population, high paraplegia often leads to erectile dysfunction and loss of ejaculatory capacity, which severely compromises natural fertility. These physiological impairments, combined with the psychological and social consequences of infertility, create substantial barriers for them to achieve biological parenthood and establishing families.

In these men, neural pathways are disrupted, leading to damages to the thoracolumbar and sacral spinal segments, which impairs the coordination of reflexogenic and psychogenic erections as well as the emission and ejaculation processes [4]. As a result, affected individuals frequently experience erectile dysfunction and loss of ejaculatory function [5], making natural conception biologically unfeasible. In addition, many patients experience significant psychological distress as the loss of reproductive function often leads to feelings of inadequacy, diminished masculinity, and concerns about long-term partnership prospects [6]. These emotional responses can be further aggravated by the perceived inability to fulfill expected life milestones such as fatherhood and forming a family, contributing to reduced self-worth and social withdrawal. Despite these profound challenges, previous studies have reported that nearly 86% of childless men with SCI still express a strong desire to have biological children and establish families [7, 8], reflecting not only the enduring importance of parenthood as a personal and social aspiration but also the necessity for medical and nursing professionals to actively address fertility concerns in this population. Thus, incorporating fertility counseling and offering viable assisted reproductive options could be essential components of comprehensive care for men with high paraplegia [9].

Recent developments in assisted reproductive technologies (ART) have greatly improved fertility options for men with high paraplegia [10]. In particular, the combination of testicular sperm aspiration (TESA) and intracytoplasmic sperm injection (ICSI) has emerged as an effective strategy to circumvent ejaculatory dysfunction by enabling the direct retrieval of viable sperm from testicular tissues, followed by fertilization via microinjection into mature oocytes [7, 11]. As a result, TESA-ICSI offers a viable pathway to biological fatherhood for patients who would otherwise face insurmountable barriers to natural conception. However, the implementation of ART in this population involves challenges that extend beyond conventional fertility care as men with high paraplegia require individualized and multidisciplinary approaches that address both technical and psychosocial aspects of reproductive treatment [12]. Therefore, comprehensive nursing care is essential, such as the inclusion of psychological assessments, evaluation of family support systems, discussions of ethical considerations regarding parenthood, and the provision of tailored education for patients and their partners throughout the reproductive process.

Despite the clinical relevance, there remains a lack of literature on nursing care protocols designed for men with high paraplegia undergoing TESA-ICSI. The complexity of their care needs necessitates specialized knowledge and interventions that extend beyond routine fertility nursing. Given their physical impairments, emotional vulnerabilities, and unique social contexts, the development of evidence-based, holistic nursing frameworks is essential to support positive reproductive outcomes and enhance patient well-being.

We hypothesize that targeted nursing interventions can improve the clinical and psychosocial outcomes of men with high paraplegia undergoing TESA-ICSI. Thus, we designed this study to evaluate the effectiveness of comprehensive care strategies in this setting, including psychological support, ethical consultation, and individualized perioperative management. By analyzing real-world cases, our results provide insights into developing specialized nursing protocols and improving fertility care for this underserved population.

## Patients and Methods

### Study design and patient selection

**Fig. 1:**
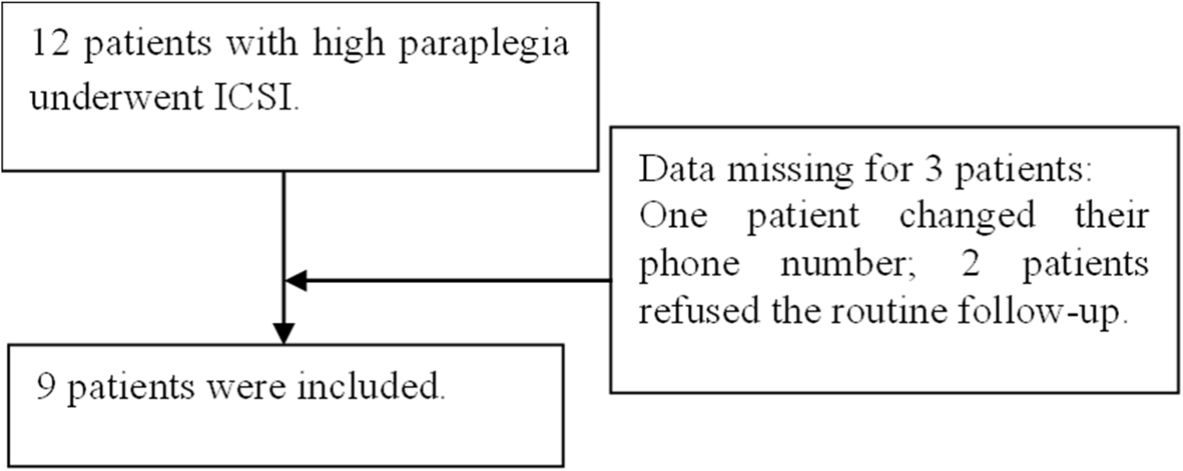
A Flowchart about patients included and excluded. (ICSI: intracytoplasmic sperm injection)

This retrospective study evaluated clinical and nursing data from couples treated at the Assisted Reproduction Unit of Sir Run Run Shaw Hospital, Zhejiang University School of Medicine (Zhejiang, China), between January 2021 and October 2023. Eligible participants included male patients diagnosed with high paraplegia due to SCI above the second thoracic vertebra (T2) who decline electroejaculation treatment, along with their female partners receiving assisted reproductive therapy. The number of included and excluded observations is shown in Fig. 1. The study was approved by our hospital’s Ethics Committee (Approval No.: 20230771), and written informed consent was obtained from all participants.

### Psychological and quality of life assessment

Before treatment, male patients completed a comprehensive psychological evaluation using the Self-rating Anxiety Scale (SAS) [13], Self-rating Depression Scale (SDS) [14], Social Support Rating Scale (SSRS) [15], and the World Health Organization Quality of Life-BREF (WHOQOL-BREF) [16] to assess their mental health status, quality of life, marital dynamics, and social support. The status of anxiety and depression were quantified as exceeding 50 points, respectively. Social support was measured using the SSRS, where scores above 6 indicated adequate support from family or community. Overall quality of life was assessed with the WHOQOL-BREF, which evaluates four domains, physical, psychological, social and environmental, on a 100-point scale, with higher scores indicating better perceived well-being.

### Ethical evaluation and reproductive suitability

Each case was reviewed by the hospital’s reproductive ethics committee to ensure responsible use of assisted reproductive technologies [17]. The evaluation considered physical function, family financial status, availability of caregiving support, spousal health and caregiving capacity, and broader implications for child welfare. These assessments guided case approval and emphasized the ethical responsibility of providers to balance reproductive autonomy with long-term parenting feasibility.

### Testicular sperm aspiration procedure

TESA was performed under local anesthesia with the patient placed in a supine position. The genital area was exposed and disinfected, and lower limbs and catheters were properly secured. A 0.5 ml dose of 1% lidocaine was administered to prevent autonomic dysreflexia, particularly in patients with elevated muscle tone (n = 4) [18]. A 20 ml syringe preloaded with culture medium was used to aspirate testicular tissue through 2 ~ 3 negative-pressure punctures (10–15 ml each). The retrieved samples were immediately examined for viable sperm under microscopy and cryopreserved for future use [19].

### Perioperative nursing care and infection prevention

Specialized nursing measures were implemented throughout the TESA process. Preoperative preparation included bladder emptying and assisted diaper changes, followed by thorough genital disinfection. After the procedure, the puncture site was compressed for 5–10 min, with hematoma checks conducted every 15 min. Patients were observed for two hours before discharge. To prevent the surgical site from becoming infected, postoperative care included a three-day course of oral antibiotics and education on wound care and urine contamination prevention.

### Ovulation induction and ICSI procedures for female partners

After successful sperm cryopreservation, ovulation induction regimens were individually tailored for each female partner based on age, ovarian reserve, hormonal profiles, and ultrasound findings. Controlled ovarian stimulation was followed by serial monitoring to determine optimal timing for oocyte retrieval. On the day of retrieval, previously cryopreserved sperm samples were thawed, assessed for motility and viability, and immediately used for ICSI. Resulting embryos were cultured under standard laboratory conditions, and either fresh or frozen embryo transfer was performed based on endometrial receptivity and patient preference.

### Supportive services for patients with mobility limitations

To accommodate mobility challenges, a streamlined service system was developed. Clinical assessments and consultations were combined into single-day visits to reduce travel burden. Reproductive education was delivered through digital platforms, and proxy consent procedures were established for patients unable to attend in person. Both partners were actively engaged throughout the process to ensure coordinated care and shared decision-making [20].

### Statistical analysis

All statistical analyses were performed using SPSS version 26.0 (IBM Corp., Armonk, NY, USA). Descriptive statistics were used to summarize patient characteristics, psychological assessments (SAS, SDS, SSRS, WHOQOL-BREF), and reproductive outcomes. Continuous variables, such as age and questionnaire scores, were expressed as mean ± standard deviation (SD). Categorical variables, including fertility outcomes and presence of psychological distress, were reported as frequencies and percentages. The relationships between psychological variables and clinical outcomes were explored descriptively due to the small sample size. A P value of < 0.05 was considered statistically significant.

## Results

### Baseline characteristics of patients with high paraplegia

A total of nine male patients with high paraplegia were included in the study. You can see specific information in Table 1.

**Table 1:** Demographic, clinical, and psychosocial characteristics of paraplegic patients included in the case series (N = 9)

No	Age	Marriage	Education	Occupation	Income	Cause of injury	Duration	Activities	WHO QOL-BREF	SSRS	SAS	SDS
P1	28	1.5	Bachelor’s degree	Athletes	5001 ~ 10,000	Traffic accident	3	Partial self-care	91	6	47	50
P2	31	1	Bachelor’s degree	E-commerce	> 10,000	Traffic accident	4	Cannot self-care	82	5	53	55
P3	42	12	Technical secondary school	Individual household	> 10,000	Traffic accident	8	Partial self-care	89	6	51	53
P4	28	2	College degree	Civil servant	5001 ~ 10,000	Traffic accident	1	Partial self-care	90	5	46	51
P5	26	2	Senior high school	Finance	5001 ~ 10,000	Traffic accident	6	Cannot self-care	85	6	50	52
P6	34	3	Senior high school	E-commerce	5001 ~ 10,000	High-altitude falling	4	Partial self-care	82	7	50	52
P7	33	1.5	College degree	E-commerce	5001 ~ 10,000	Traffic accident	2	Cannot self-care	90	5	45	49
P8	35	1	Bachelor’s degree	No	> 10,000	Trauma	13	Partial self-care	88	6	49	50
P9	30	0.5	Bachelor’s degree	No	5001 ~ 10,000	Spinal cord arterial occlusion	1.5	Cannot self-care	86	5	56	55

a Age(years)

b Marriage(Years of marriage)

c Education(Educational attainment)

d Income(Monthly household income: RMB. < 5000 RMB = poor, 5001 ~ 10,000 RMB = moderate, > 10,000 RMB = rich)

e Duration( Duration of paralysis)

f Activities (Activities of daily living)

g WHO QOL-BREF(World Health Organization Quality of Life-BREF)

h SSRS(Social Support Rating Scale)

i SAS(Self-rating Anxiety Scale)

j SDS(Self-rating Depression Scale)

k N/No.(number)

### Psychological and Social Assessment Outcomes

Psychological and quality of life assessments revealed the following: SAS scores ranged from 45 to 56, with three patients scoring ≥ 50, indicating clinically relevant anxiety (Table 1). SDS scores ranged from 49 to 55, with four patients scoring ≥ 53, suggesting depressive symptoms. SSRS scores ranged from 5 to 7, with eight patients scoring ≥ 6, reflecting moderate to good levels of social support. WHOQOL-BREF scores ranged from 82 to 91, indicating generally good perceived quality of life.

### Perioperative sperm retrieval and safety evaluation

All nine patients successfully underwent TESA. Four patients received local anesthesia due to increased muscle tone, although none experienced pain owing to the sensory deficits associated with high paraplegia. Intraoperative monitoring confirmed successful aspiration in all cases, and available spermatozoa were identified and cryopreserved for future use.

Preoperative preparation included bladder emptying and perineal disinfection, and postoperative management involved compression of the puncture site for 5–10 min, antibiotic prophylaxis for 3 days, and observation for hematoma formation every 15 min. No cases of infection or hematoma were recorded. All patients remained hemodynamically stable and were discharged after a 2-h observation period.

### Assisted reproductive treatment and embryo transfer outcomes

Among the nine couples enrolled, eight female partners (88.9%) completed the full ICSI-assisted reproductive protocol. Viable cleavage-stage were successfully obtained in all eight cases. Among these, 6 couples (66.7%) achieved clinical pregnancy following embryo transfer. To date, all 6 pregnancies have resulted in successful live births(underwent transplantation of cleavage-stage embryos and blastocysts, respectively). Of the remaining 2 cases, 1(11.1%) experienced an inevitable miscarriage due to accidental trauma, while the other (11.1%) failed to develop blastocyst-stage. Among the couples who completed embryo transfer, the live birth rate was 66.7%.

## Discussion

This study highlights that specialized, multidisciplinary nursing interventions can facilitate the successful application of TESA combined with ICSI in male patients with high paraplegia. A live birth rate of 66.7% is comparable to the findings reported in studies involving healthy populations[21]. Our findings highlight that, with tailored perioperative management, psychological support and ethical evaluation, these patients can achieve favorable reproductive outcomes, underscoring the potential feasibility and effectiveness of integrating reproductive care into the clinical management of individuals with severe SCI.

The inclusion of standardized psychological and social assessments enabled a more nuanced understanding of each patient’s mental health status and support systems. While several patients presented with moderate levels of anxiety and depression, the majority demonstrated sufficient social support and relatively high quality of life scores. In a separate study, Chen et al. reported that 56.8% of SCI patients experience significant negative emotions, including depression, anxiety, and feelings of inferiority [22]. These evaluations provide a foundation for targeted psychological support and reinforced patient eligibility for assisted reproduction. The comprehensive assessment approach employed in our study enabled targeted psychological support interventions, potentially contributing to the favorable treatment outcomes observed.

Ethical evaluation is an essential component of care, particularly for patients who were completely dependent on others for daily living. The reproductive ethics committee assessed the suitability of each case based not only on the technical feasibility of ART but also on the family’s caregiving capacity, financial stability, and the spousal partner’s willingness and ability to co-parent [23]. Such individualized ethical review helped ensure that assisted reproductive treatment was aligned with the long-term welfare of both the parents and potential offspring. Thus, the protocol established a framework for ethical decision-making that respects patient autonomy while acknowledging the complex social dimensions of parenthood in SCI [24].

Technically, the TESA procedure was successful in all nine patients, with no perioperative complications observed. This high success rate, combined with the absence of infection or hematoma, supports the safety of TESA in high paraplegia patients when appropriate procedural adaptations and nursing protocols are in place. Our findings corroborate those of Kanto et al. [25], who demonstrated comparable sperm retrieval rates between able-bodied patients and those with neurological impairments when appropriate procedural modifications are implemented. The absence of post-procedural complications in our cohort highlights the effectiveness of specialized nursing interventions, including the use of local anesthesia to prevent autonomic dysreflexia, careful positioning, and stringent aseptic precautions for minimizing risks, thereby further supporting that specialized nursing interventions may not only be supportive but also integral to the procedural success of sperm retrieval in SCI patients.

Encouraging reproductive outcomes further supports the utility of this approach. Among the eight couples who completed embryo transfer, six achieved pregnancy, including four deliveries and two ongoing pregnancies beyond 28 weeks, resulting in a potential cumulative success rate of 75%. These outcomes compare favorably with previous reports in SCI populations, such as that of Iwahata et al. [7], who reported clinical pregnancy rates of 35–45% after TESA-ICSI in their cohort of SCI patients. In our study, the promising results could be attributed to early identification of eligible candidates, consistent spousal involvement, and coordinated nursing support throughout the assisted reproductive cycle. The proactive engagement of female partners and their understanding of the process through digital platforms and in-clinic guidance also contributed to improved adherence and emotional resilience during treatment.

Our findings also demonstrate the importance of integrating family support systems into the care model for men with high paraplegia. All patients in our cohort reported stable family relationships and adequate financial resources, which likely contributed positively to treatment outcomes. This observation aligns with research by Jeyathevan et al. [12], who identified family support as a key determinant of psychological adaptation and quality of life among individuals with SCI [24]. The implementation of fast-track services and digital education resources further facilitated family involvement throughout the treatment process [26].

Moreover, this study highlights the necessity of early fertility preservation counseling for young men with SCI. As reported by Krebs et al. [20], sperm quality in men with SCI tends to decline over time, suggesting that early sperm cryopreservation may optimize future reproductive potential. Our findings support recommendations from the Chinese Expert Consensus on Male Fertility Preservation [27], which advocates for proactive fertility discussions and preservation options immediately following SCI diagnosis in young male patients.

Taken together, our findings suggest that men with high paraplegia can achieve biological parenthood through ART when supported by comprehensive, ethically grounded, and technically adapted nursing interventions. This model of care not only facilitates successful clinical outcomes but also addresses the broader psychosocial and ethical challenges inherent to reproductive treatment in patients with severe physical disabilities. It reinforces the need for specialized, interdisciplinary care protocols to support the reproductive rights and well-being of this underserved population.

### Limitations

There were several limitations that should be acknowledged. The small sample size (n = 9) and retrospective design restrict the generalizability of the findings, and the single-center setting with access to specialized resources may not reflect the conditions of other healthcare environments. Additionally, long-term follow-up data on children born through this program were not included, limiting insight into postnatal outcomes. Future research should include prospective, multicenter studies with larger sample sizes to validate these results and identify additional factors influencing reproductive outcomes in men with high paraplegia. Further investigation into the long-term psychological adjustment of both patients and their offspring would enhance understanding of the broader implications of assisted reproduction in this context. Moreover, the development and implementation of standardized nursing protocols tailored to the needs of SCI patients undergoing fertility treatment are warranted to improve care consistency and quality across institutions.

## Conclusion

This retrospective study confirms that men with high paraplegia can attain biological fatherhood via TESA and ICSI with specialized nursing care. A comprehensive approach—including psychological assessment, ethical review, procedural adaptations, and family engagement—was key to favorable outcomes. Success rates show paraplegia does not inherently limit reproductive potential with proper support, underscoring the need for standardized nursing protocols and interdisciplinary collaboration to deliver safer, tailored fertility care for this group.

## Data Availability

The datasets analyzed in this study are not publicly available at present because we are still using the data for another research.However, they can be obtained from the corresponding author upon reasonable request.

## Abbreviations

ART: Assisted reproductive technologies
ICSI: Intracytoplasmic sperm injection
NTSCI: Non-traumatic SCI
SAS: Self-rating Anxiety Scale
SCI: Spinal cord injury
SD: Standard deviation
SDS: Self-rating Depression Scale
SPSS: Statistical Package for the Social Sciences
SSRS: Social Support Rating Scale
TESA: Testicular sperm aspiration
TSCI: Traumatic SCI
T2: The second thoracic vertebra
WHOQOL-BREF: World Health Organization Quality of Life-BREF
